# Compact automated culture machine for human induced pluripotent stem cell maintenance and differentiation

**DOI:** 10.3389/fbioe.2022.1074990

**Published:** 2022-11-29

**Authors:** Kazunori Bando, Hiromi Yamashita, Motomu Tsumori, Hayase Minoura, Koji Okumura, Fumiyuki Hattori

**Affiliations:** ^1^ Innovative Regenerative Medicine, Kansai Medical University Graduate School of Medicine, Osaka, Japan; ^2^ New Business Promotion Center, Panasonic Production Engineering Co., Ltd., Osaka, Japan

**Keywords:** keratinogenic differentiation, neurogenic differentiation, hepatogenic differentiation, cardiogenic differentiation, human induced pluripotent stem cell, automated culture machine

## Abstract

The technologies used to generate human induced pluripotent stem cell (iPSC) from somatic cells potentially enable the wide application of iPSC-derived differentiated cells in industrial research fields as a replacement for animals. However, as highly trained individuals are required to obtain reproducible results, this approach has limited social implementation. In the research field of iPSC, it is believed that documentable information is not enough for reproducing the quality of the differentiated cells. Therefore, automated culture machines for cell processing should make the starting of iPSC-using researches easier. We developed a programmable all-in-one automated culture machine, with dense and compact constitution that fits within a normal biosafety cabinet (200 mm wide, 233 mm height, and 110 mm depth). This instrument was fabricated using novel x-y-z-axes-rail-system, such as an overhead traveling crane, in a factory, which served as the main handling machinery. This machine enabled stable and efficient expansion of human iPSC under the feeder-free condition, without karyotype alterations, and simultaneously differentiated the cells into various cell types, including cardiomyocytes, hepatocytes, neural progenitors, and keratinocytes. Overall, this machine would facilitate the social implementation of human pluripotent stem cells and contribute to the accumulation of sharable knowledge for the standardization of the entire handling processes of iPSC in pharmaceutical, food, and cosmetic research.

## 1 Introduction

Human induced pluripotent stem cell (iPSC) can infinitely supply various types of non-immortalized human somatic cells for industrial research. In accordance with advancements and the generalization of ethics for animal welfare, in principle, animal experiments must be avoided. Therefore, alternative cell-based experimental systems are highly required. Although human iPSC appears to be the most hopeful and ideal cell source for such purposes, it has not been generally used in the pharmaceutical, food, and cosmetic industries; this is due to the difficulties associated with handling iPSC, which is highly sensitive to artificial factors and environmental alterations (Bertero et al., 2015; Wang et al., 2017; Stavish et al., 2020; Keller et al., 2018).

Several automated culture systems have been developed for iPSC (Sasamata et al., 2021; Tristan et al., 2021). The systems that can perform maintenance and differentiation are equipped with human-like multi-articulated arm(s). Although such systems can mimic human actions, they require larger spaces and advanced teaching technique and are associated with higher costs. Among them, the most unfavorable feature of this system is associated with a new operation, as enormous time and effort must be dedicated to optimize the combinations of all joint movements at the right time, angle, and speed (Gonzalez and Zalewski, 2017). To avoid the above disadvantages, we adopted a completely different strategy to achieve the same ability with humanized multi-articulated arms. Here, we demonstrated the efficient maintenance and differentiation of human iPSC using this machine.

## 2 Materials and methods

### 2.1 Human induced pluripotent stem cells

The hiPSC lines, RIKEN-2F and 253G1, were obtained from the Laboratory for Pluripotent Cell Studies, RIKEN Center for Developmental Biology, Tsukuba city, Ibaragi, Japan. The original iPSC line named “KMUR001” was established using healthy volunteer’s blood as described elsewhere (Okita et al., 2011).

### 2.2 Human induced pluripotent stem cells maintenance

#### 2.2.1 Detachment and single-cell dispersion of induced pluripotent stem cells in the culture plate

The hiPSC lines were maintained in 10 cm plastic plates (Corning Inc., NY, United States) coated with 0.5 μg/cm^2^ iMatrix511 Silk (Nippi Inc., Tokyo, Japan), with Neutristem^®^ (Sartrius AG, Göttingen, Germany) or StemFit^®^ AK02N (Ajinomoto, Tokyo, Japan) as the culture medium. Cells attached to the plate were washed three times with each 5 ml phosphate buffer saline without calcium (PBS(-); Nacalai Tesque Inc., Kyoto, Japan) with three sets of three-times cross-rocking movements in each wash process, and treated with 3 ml TripLE™ express enzyme (Thermo Fisher Scientific, Waltham, MA, United States) supplemented with 10 μM of the Rho kinase inhibitor, Y-27632 (Sellec Inc., Tokyo, Japan), for 15 min at 37°C. Detachment of all cells from the plate and dispersion into single cells were performed as described below. First, 9 ml of PBS(-) supplemented with 0.15% bovine serum albumin fraction V (BSA: Fuji Film Wako Chemical Inc., Miyazaki, Japan) was added to the plate. Thereafter, 10 ml of the aspirated cell-containing solution was vigorously dispensed from the pipette tip close to the plate-plane, with a zig-zag motion around the upper-side of the 20° tilted-plate. The machine performed this twice. Then, the culture plate was tilted −20° and the above the same process was repeated. The above set of the movements was sequentially performed three-times. Thereafter, the aspiration and dispensing of cell-containing medium was performed twice along the side-wall of the plate for each 20°, and −20° tilted-plate. Finally, all cells were collected in a 15-ml tube, capped, and centrifuged at 800 rpm (115 x *g*) for 5 min to deposit the cells. The supernatant was then aspirated, and the cell pellet was dispersed into single cells using 10 ml Neutristem or Stemfit supplemented with 10 μM Y-27632 *via* 10 rounds of pipetting.

#### 2.2.2 Cell counting

One milliliter of trypan blue solution (Nacalai Tesque, Kyoto, Japan) was transferred into a new 15-ml tube. Thereafter, 1 ml of the cell suspension solution was added and mixed well *via* five rounds of pipetting. One hundred microliters of this trypan blue-stained cell solution was aspirated using a pipette and dispensed into the entry window of a disposable hemocytometer (Countess™ Cell Counting Chamber Slides, Thermo Fisher Scientific) in the hemocytometer holder. Then, the hemocytometer holder was moved to the microscopic observation area. The captured image was immediately analyzed using the software; and the obtained cell concentration was applied to the next process(es).

#### 2.2.3 Re-seeding of induced pluripotent stem cell in 10 cm diameter and/or 6-well plates

For maintenance of iPSC, the cells were seeded in 10 cm diameter plastic plate(s) (Corning Inc., NY, United States) coated with 0.5 μg/cm^2^ iMatrix511 Silk (Nippi Inc., Tokyo, Japan) at a cell density of 15,000 cells/cm^2^. For the differentiation experiments, the cells were seeded in 6-well plates coated with iMatrix511 or the 1/100-diluted Matrigel^®^ (Corning Inc.) at the required cell density with the prepared medium for each somatic cell type. Five-times cross-rocking was performed before the cells were transferred to the incubator. The digested movement of the machine in the above “passage task” is shown in Supplementary Movie S1.

### 2.3 Immunohistochemistry

Manually, the cells were fixed in 4% paraformaldehyde for 5 min at 25°C. Thereafter, the cells were washed twice with Tris-buffered saline containing 0.2% Tween-20 (TBS-T) and treated with a blocking solution (Nacalai Tesque) for 30 min at 25°C. The first antibody-containing blocking agent was added to the cells and incubated overnight at 4°C with paraffin sealing to prevent evaporation. The cells were washed thrice with TBS-T and immersed in the second antibody-containing blocking agent for 1 h at room temperature. After three washes, the fluorescent signals were observed using a fluorescence microscope (Nikon Instruments, Tokyo, Japan). The primary and secondary antibodies used are listed in Supplementary Table S1.

### 2.4 Immunocytometorical analysis

After the automatic passage procedure was performed by the machine, the residual iPSCs were manually retrieved from the machine and fixed with 4% paraformaldehyde for 5 min at 25°C. After centrifugation, the supernatant was aspirated and dispensed into the waste bottle for appropriate disposal. The fixed cells were washed twice with TBS-T, and then treated with blocking reagent (Blocking One; Nacali Tesque) for 30 min at 37°C. The cells were divided into three groups, and each group was treated with no primary antibody or primary antibody against Oct-3/4 or SSEA4 at 4°C for 18 h. After the cells were washed three times with TBS-T, they were treated with the secondary antibody at 25°C for 30 min. The stained cells were analyzed using FACS Aria™ III (BD Biosciences, Franklin Lakes, NJ, United States). As the common gating strategy, to eliminate doublets, the major FSC-A and SSC-A populations were gated by FSC-H and -W, followed by SSC-H and -W. The data obtained from the cells treated with the secondary antibody served as the negative control. Data of the 50,000 events were obtained using each sample.

### 2.5 Differentiation

#### 2.5.1 Cardiomyocytes

Undifferentiated hiPSC (KMUR001) were seeded at a density of 30,000 cells/cm^2^ in a 6-well plate coated with iMatrix511 Silk in StemFit supplemented with 10 μM of the Rho kinase inhibitor, Y-27632, 2 days before the first day of differentiation. On day 1, the medium was changed to Essential 8 (Thermo Fisher Scientific) supplemented with 6 μM of the GSK-3ß inhibitor, CHIR-99021 (MCE, NJ, United States #HY-10182), 20 ng/ml Activin A (Nacali Tesque), and 10 ng/ml BMP4 (Thermo Fisher Scientific). On differentiation day 3, the medium was changed to CDM3 medium: RPMI 1640 (#11875, Thermo Fisher Scientific), 500 μg/ml recombinant human albumin (A0237, Merck, Darmstadt, Germany), and 213 μg/mL L-ascorbic acid 2-phosphate (A8960, Merck) supplemented with 2 µM Wnt-C59 (Bio-techne, NB, United Kingdom). On differentiation day 5, fresh CDM3 medium was added. Thereafter, fresh CDM3 medium was added every other day.

#### 2.5.2 Hepatocytes

Hepatic differentiation from hiPSC (RIKEN2F) was performed as described previously, with some modifications. Human iPSCs were passaged and then seeded at a cell density of 25,000 cells/cm^2^ on iMatrix511-coated 6-well plate in Stemfit^®^ and cultured for 2 days. Subsequently, to initiate differentiation, the cells were treated with RPMI-1640 plus 2% B27 Minus Insulin (RPMI-B27) containing 100 ng/ml Activin A (Nacali Tesque), 6 µM CHIR-99021 (MCE), and 1% GlutaMAX^®^ (Thermo Fisher Scientific) for 24 h, followed by RPMI-B27 with 50 ng/ml Activin A for 24 h. For differentiation into hepatic progenitors, the cells were treated with RPMI-B27 containing 1% GlutaMAX^®^ and 10 ng/ml BMP-4 (Thermo Fisher Scientific) for 4 days. The medium was changed to hepatocyte maturation medium: Leibovitz’s L-15 medium (Fuji Film Wako Chemical Inc.) containing 8.3% tryptose phosphate broth, 10 μM hydrocortisone 21-hemisuccinate, 50 μg/ml sodium L-ascorbate, 100 nM dexamethasone (DEX) (all from Merck), 0.58% insulin-transferrin-selenium (ITS), 2 mM GlutaMAX^®^ (all from Thermo Fisher Scientific), 8.3% fetal bovine serum (Biowest, FL, United States), and 100 nM Dihexa (TRC, Ontario, Canada). This culture was continued for 17 days to obtain definitively differentiated hepatocyte-like cells. During the culture, media change was performed every 2 days.

#### 2.5.3 Neuronal precursor cells

For the initiation of differentiation (Day 1), undifferentiated iPSC (RIKEN2F) were seeded at a density of 25,000 cells/cm^2^ on a 6-well plate coated with Matrigel in Dulbecco’s modified Eagle medium (DMEM)/F12 (Fuji Film Wako Chemical Inc.) and Neurobasal medium (1:1; Thermo Fisher Scientific) supplemented with 10% Knockout serum replacement (Thermo Fisher Scientific), 0.1 mM non-essential amino acids, 1 mM GlutaMAX^®^ with 10 μM TGF-beta receptor inhibitor (SB431542), 10 μM BMP signal inhibitor (dorsomorphin), and 10 μM Y-27632. Media change was performed on day 3. On differentiation day 5, the medium was changed to DMEM/F12 and Neurobasal medium 1:1 supplemented with 0.1 mM non-essential amino acids, 1 mM GlutaMAX^®^, 1% N-2 supplement, 1% B-27 supplement (all from Thermo Fisher Scientific), 50 μg/ml ascorbic acid 2-phosphate (Merck), 10 μM SB431542, and 10 μM dorsomorphin. Media change was performed on days 7 and 8. On differentiation day 9, the medium was changed to DMEM/F12 and Neurobasal medium 1:1 supplemented with 0.1 mM non-essential amino acids, 1 mM GlutaMAX^®^, 1% N-2 supplement, 1% B-27 supplement, 50 μg/ml ascorbic acid 2-phosphate, and 1 μM all-trans retinoic acid. Media change was performed every day. From differentiation day 13–16, media change was performed every day with DMEM/F12 and Neurobasal medium 1:1 supplemented with 0.1 mM non-essential amino acids, 1 mM GlutaMAX^®^, 1% N-2 supplement, 1% B-27 supplement, 50 μg/ml ascorbic acid 2-phosphate, and 10 ng/ml bFGF and 10 ng/ml EGF.

#### 2.5.4 Keratinocytes

Undifferentiated hiPSC (253G1) were seeded at a density of 15,000 cells/cm^2^ in a 6-well plate coated with iMatrix511 Silk using StemFit supplemented with 10 μM Y-27632, 2 days before the first day of differentiation (day 1). To initiate the differentiation, the medium was changed to Dulbecco’s modified Eagle medium (DMEM)/F12 (Fuji Film Wako Chemical Inc.) and Neurobasal medium (1:1; Thermo Fisher Scientific) supplemented with 0.1 mM non-essential amino acids, 1 mM glutamine, 55 μM 2-mercaptoethanol, 1% N-2 supplement, 2% B-27 supplement (all from Thermo Fisher Scientific), 50 μg/ml ascorbic acid 2-phosphate (Merck), 0.05% bovine serum albumin (Merck), and 100 ng/ml FGF-basic (Nacalai Tesque Inc.). On day 3 and thereafter, the medium was changed to Defined Keratinocyte-SFM (without addition of the attached supplement for day 3 and with the supplement on/after day 5; Thermo Fisher Scientific) with addition of 0.5 μg/ml hydrocortisone, 1 μM all-trans retinoic acid, 25 ng/ml hBMP-4 (Thermo Fisher Scientific), 2.4 μg/ml adenine, 1.37 ng/ml triiodothyronine, 0.3 mM ascorbic acid 2-phosphate, and 2 μM forskolin, every 2 days. Between days 4 and 5, the cells formed self-organized aggregates, and some re-attached to the plate after a few days.

### 2.6 Ethics

The Ethics Committee of the Kansai Medical University approved the generation and use of the healthy volunteer-derived iPSCs named KMUR001 (approval No. 2020197). The donor, who was openly recruited, provided formal informed consent and agreed with the scientific usage of the cells.

## 3 Results

### 3.1 Design and functions of the automatic culture machine

**FIGURE 1:**
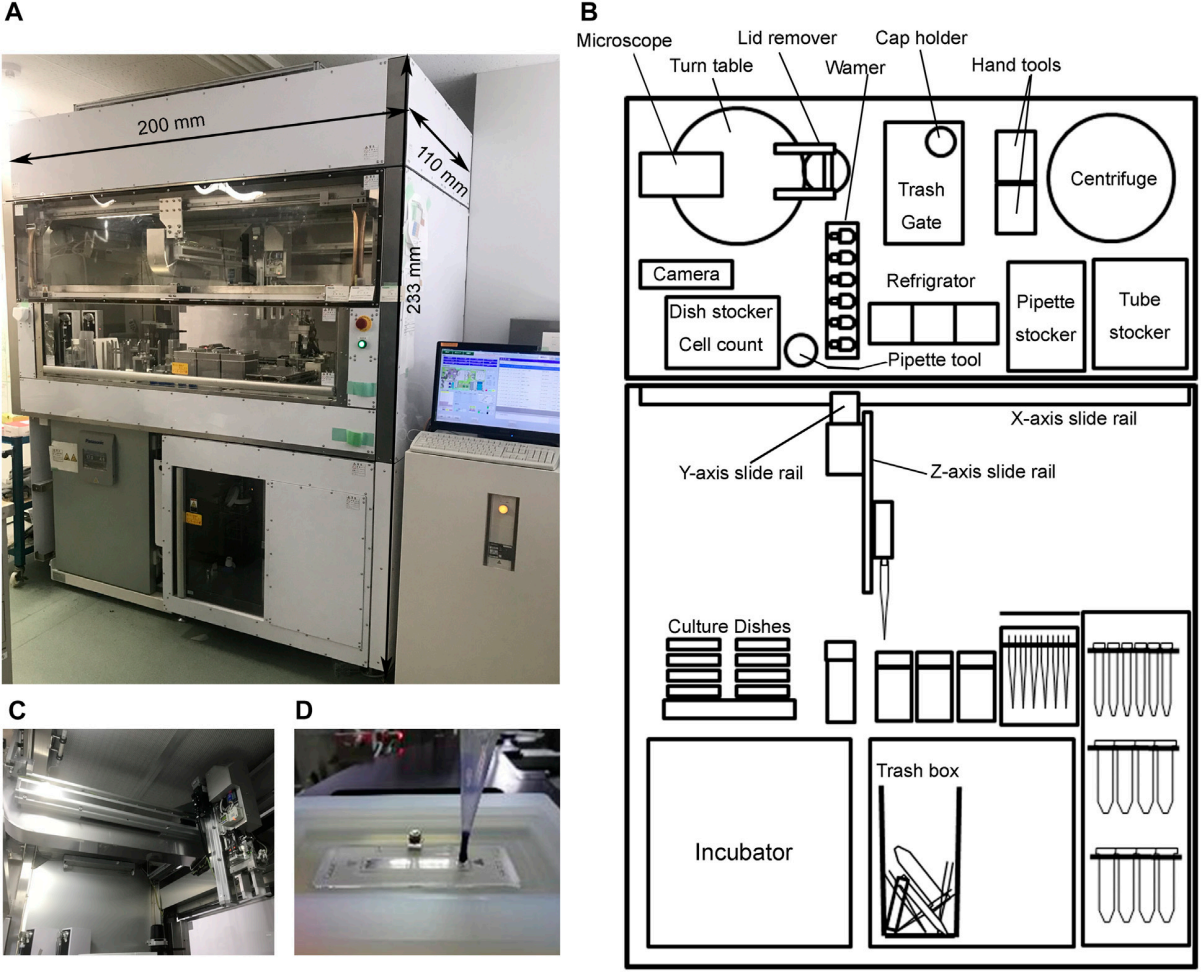
Summary of the automated culture machine. **(A)** Image of the whole machine and its size. **(B)** Schematic of the machine. **(C)** Close view of the x-y-z-axes-rail-system. **(D)** Loading of the trypan blue-stained cells into the cell counting plate.

The original machine design is illustrated previously (Konagaya et al., 2015). Notably, critical changes were applied to the design. First, the cell-/medium-/plate-handling apparatus was changed from the 6-axes articulated-robotic arm to the x-y-z axes rail system, which comprised horizontally crossed rails on the ceiling frames and vertically movable multifunctional-apparatus (Supplementary Figure S1). The multifunctional apparatus can choose three types of functional parts, including pipette-, tube-, and plate-handling tools. Current software setting of our machine is optimized to handle culture-ware formats of 10-cm dishes and 6-well plates, and can simultaneously maintain 36 dishes or nine plates. However, our machine holds ability to handle 12, 24, and 96 well plates with the same medium change-speed 70 s per well by proper modification of the software settings. Most functions of the apparatus are powered by the accumulated air pressure in the tank. As the result, the total weight of the machine is 1.2 t, which is markedly less than that of the previous version (1.4 t) (Konagaya et al., 2015). Approximately 200 kg loss of ceiling weight enabled the simpler cabinet frame constructions, which ultimately allowed the adoption of transparent sliding doors, such as ordinal biosafety cabinet in the front and back, thereby markedly improving the visibility of the machine movements and accessibility for part maintenance (Figures 1A−C).

To carry out feeder-free cultivation of human iPSC, the machine can measure cell concentration automatically. The single cell suspension solution is mixed with trypan blue solution and transferred to a disposable hemocytometer for observation *via* microscopy (Figures 1B,D). The obtained images are then analyzed and calculations are automatically performed by the software. The live cell counts obtained automatically were consistent with those obtained manually (Supplementary Figure S2).

Scheduling the culture task(s) using the original software can be done by selecting appropriate task(s) and setting the starting day/time, for example, to instruct the machine to perform cell passage from plate to plate(s), the appropriate pre-program “passage task” must be selected and the parent and the daughter plate(s) must be assigned. If necessary, the selected “task” can be modified easily by changing the parameters of time, volume, velocity, etc., without changing the parameters of original “task”.

### 3.2 Maintenance of human induced pluripotent stem cell

**FIGURE 2:**
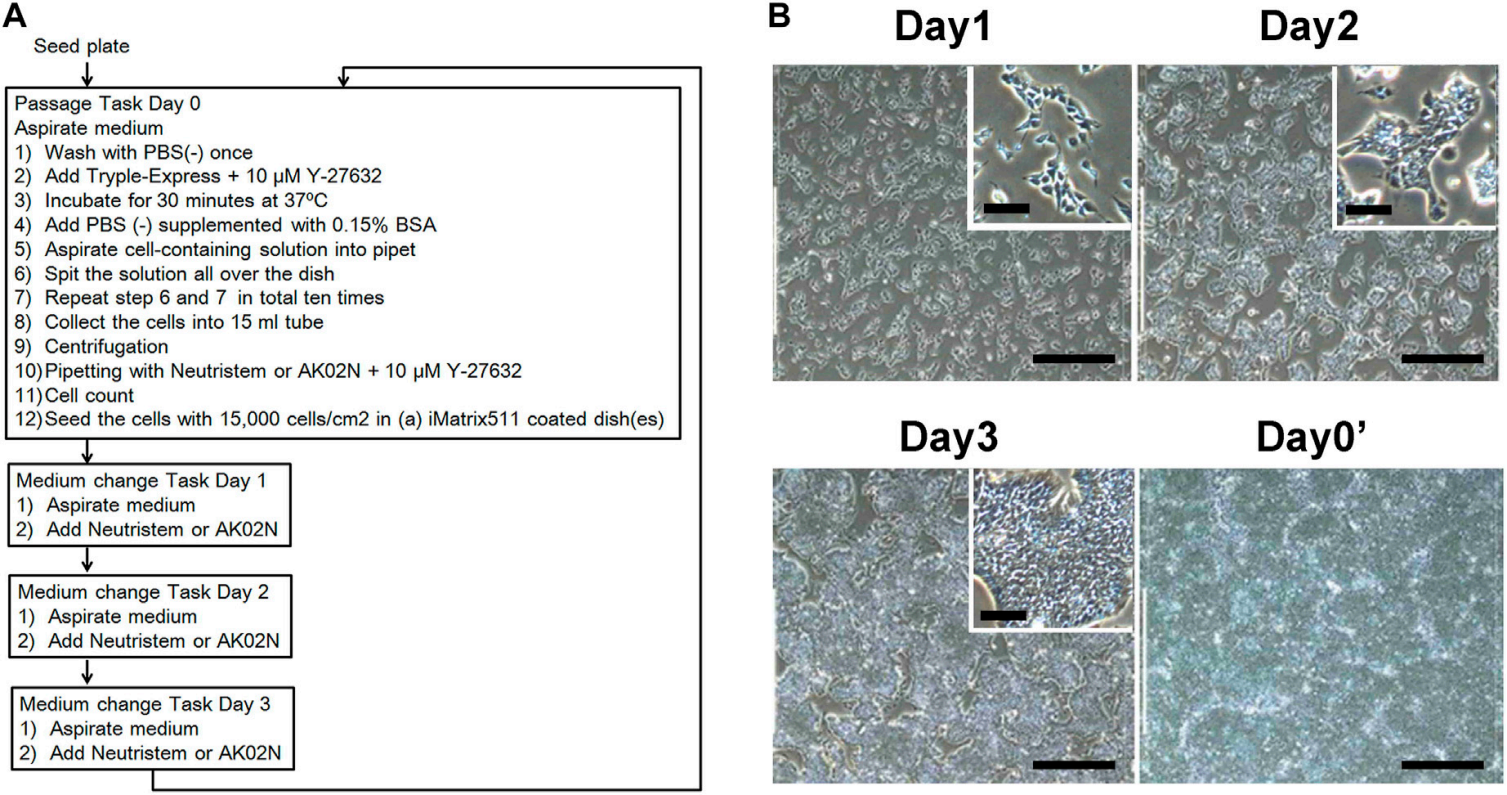
Passage task and cell growth. **(A)** The square illustrates each process. **(B)** Representative images captured automatically from passage to passage. Each inset indicates enlarged image. Scale bars, 400 μm (B); and 100 μm (inset B).

**FIGURE 3:**
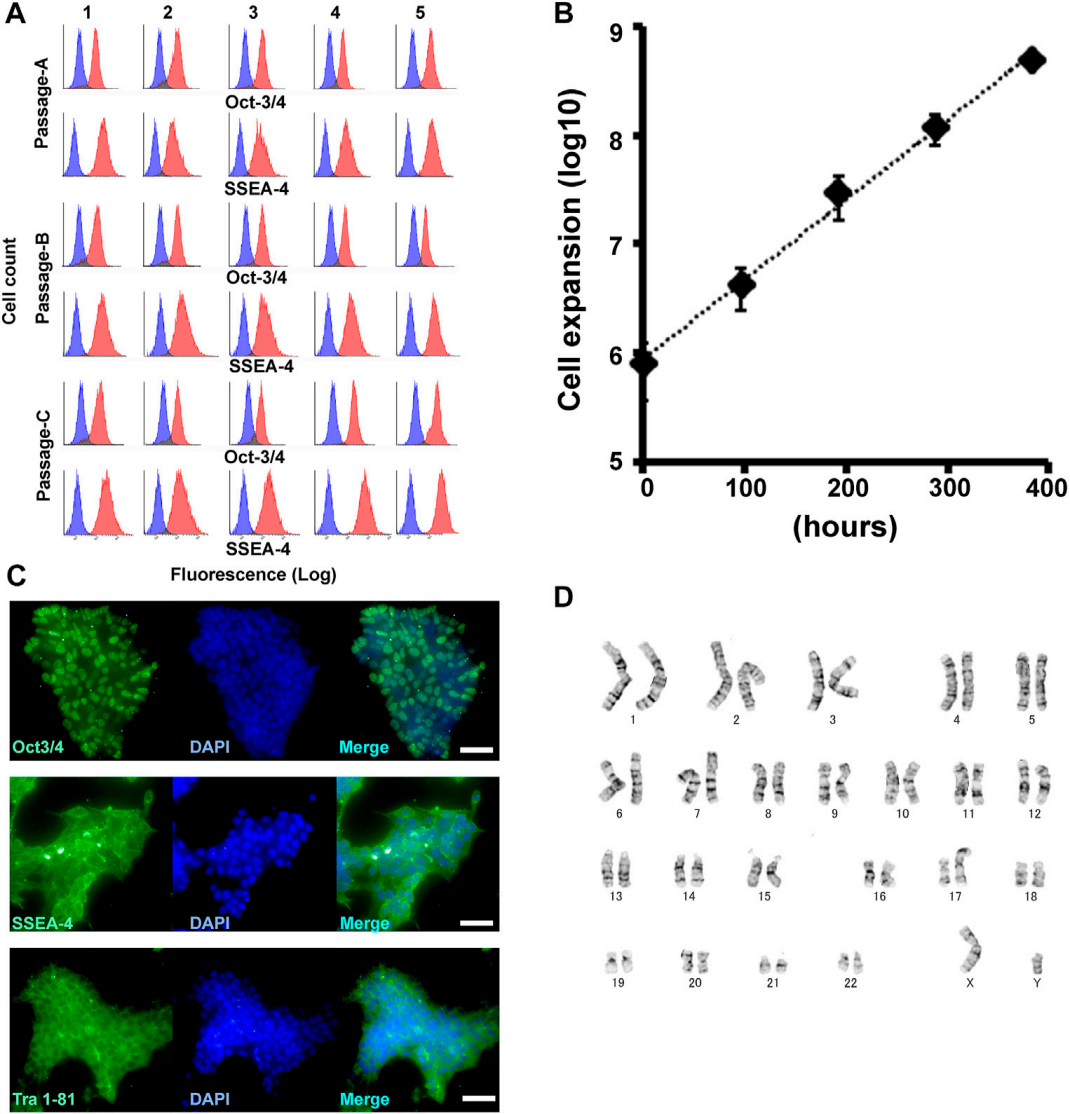
Automated long-term maintenance of iPSC. **(A)** All immunocytometorical analyses of the three independent experiments for Oct-3/4 and SSEA-4. Each blue histogram indicates no primary antibody control. Each red histogram represents the indicated antigen-specific signal. **(B)** iPSC expansion calculated using every automated cell count in the three independent experiments. **(C)** Representative images of immunohistochemically stained cells for Oct-3/4, SSEA-4, and Tra 1–61. Scale bars = 50 μm. **(D)** Karyotype of the RIKEN2F-iPSC after the fifth passage.

Triplicate 10 cm plates with the iPSC (RIKEN2F) and triplicate iMatrix-511 pre-coated plates were loaded into the machine’s humidified CO_2_ incubator and stored until use. According to the ordered schedule, the machine sequentially performed the passage procedures listed in Figure 2A for the triplicate culture plates. After 24, 48, 72, and 96 h, the culture medium in each plate was changed automatically. The coated culture plates, media, reagents, and expendables were supplied to the machine manually, if necessary, until the next scheduled passage. Five sequential passages were stably performed. Representative day by day images of growing cells are shown in Figure 2B. The residual cells after each passage were manually removed and immunocytochemical analyses were performed. Accordingly, stable maintenance of the cell pluripotency was confirmed (Figure 3A). Cell expansion (*n* = 3) was calculated and shown in Figure 3B. The human iPSCs (RIKEN 2F line) passaged five times by the machine were immunohistochemically analyzed for Oct-3/4, SSEA-4, and Tra 1–81, and their pluripotency was confirmed. We also confirmed their internal alkaline phosphatase activity by formazan production from 5-bromo-4-chloro-3-indolyl phosphate (Supplementary Figure S3). The representative features are shown in Figure 3C. The iPSC retained the original karyotype (Figure 3D). Karyotype analysis using the earliest RIKEN2F cell-stock (before five-times machine passages) revealed an abnormality in an allele of chromosome-17 and this preserved till the end of machine-passages. We compared required times for each process with those of our previous model machine (with 6-axes articulated-robotic arm). The current machine can process much faster, although total time spent in passaging is still longer than that of human operators. By contrast, the time required for cell-counting performed by the machine is shorter than by human operators due to the machine’s superior image processing ability (Supplementary Table S2). We preliminarily performed more than five sequential passages using other iPSC lines including 253G1 and KMUR001 using essentially the same task-program altered to perform passaging every 3 days.

### 3.3 Differentiation into various types of somatic cells in three germ layers

**FIGURE 4:**
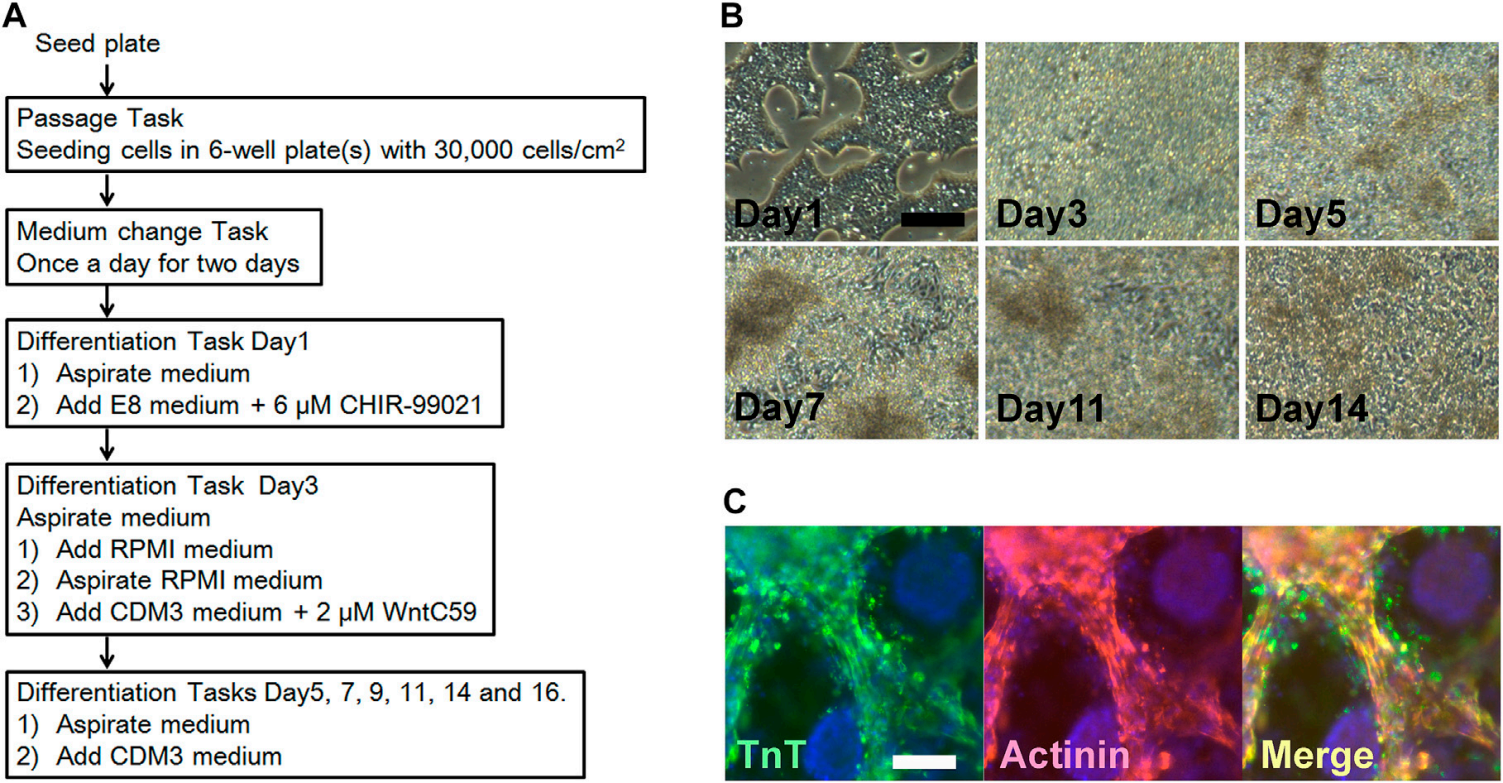
Cardiomyogenic differentiation using human iPSC (KMUR001). **(A)** Schedule of the combination of processes including passage and differentiation. **(B)** Step by step changes in the cell figures based on phase contrast micrography. Scale bar = 200 μm. **(C)** Immunohistochemical staining for Troponin T (TnT): green, and Actinin: red. The nucleus was stained with DAPI. Scale bar = 100 μm.

**FIGURE 5:**
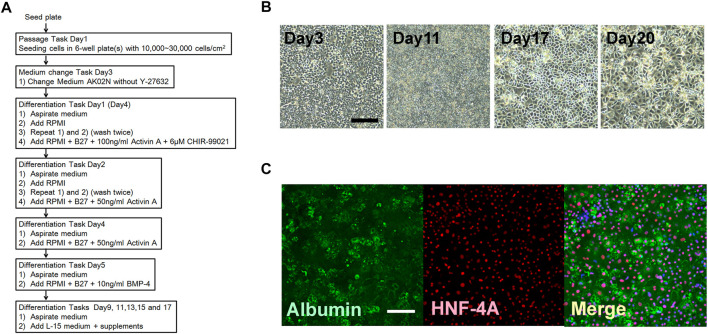
Hepatocyte differentiation using the human iPSC (RIKEN2F). **(A)** Schedule of the combination of processes including passage and differentiation. **(B)** Step by step changes in the cell figures based on phase contrast micrography. Scale bar = 100 μm. **(C)** Immunohistochemical staining for Albumin: green, and hepatocyte nuclear factor 4 alpha (HNF-4A): red. The nucleus was stained with DAPI. Scale bar = 100 μm.

**FIGURE 6:**
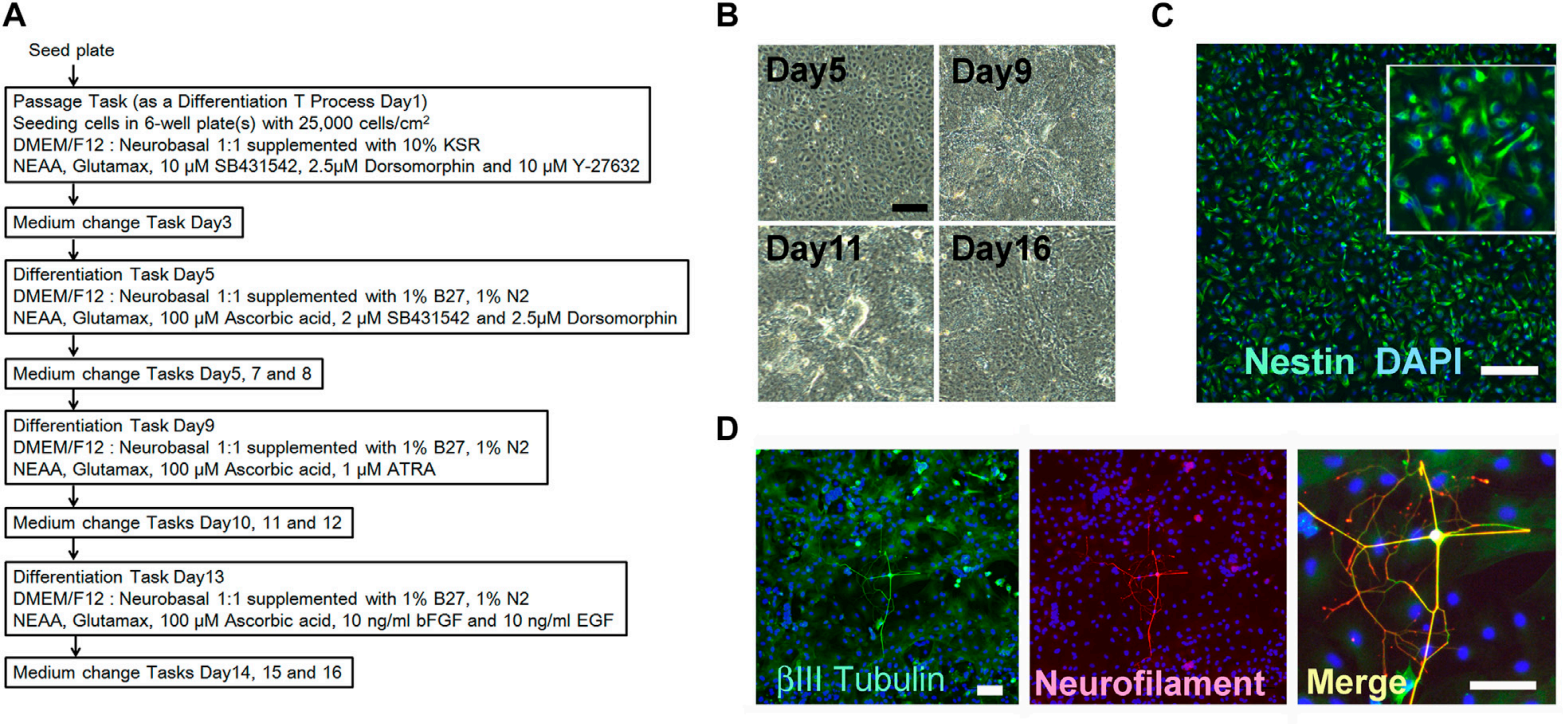
Neuronal progenitor cell differentiation using the human iPSC (RIKEN2F). **(A)** Schedule of the combination of processes including passage and differentiation. **(B)** Step by step changes in the cell figures based on phase contrast microscopy. Scale bar = 100 μm. **(C)** Immunohistochemical staining for Nestin: green. The nucleus was stained with DAPI. Scale bar = 200 μm. **(D)** Continuous culture confirmed the neuronal differentiation of the progenitor cells. Immunohistochemical staining for ßIII Tubulin: green, and Neurofilament M: red. The nucleus was stained with DAPI. Scale bar = 100 μm.

**FIGURE 7:**
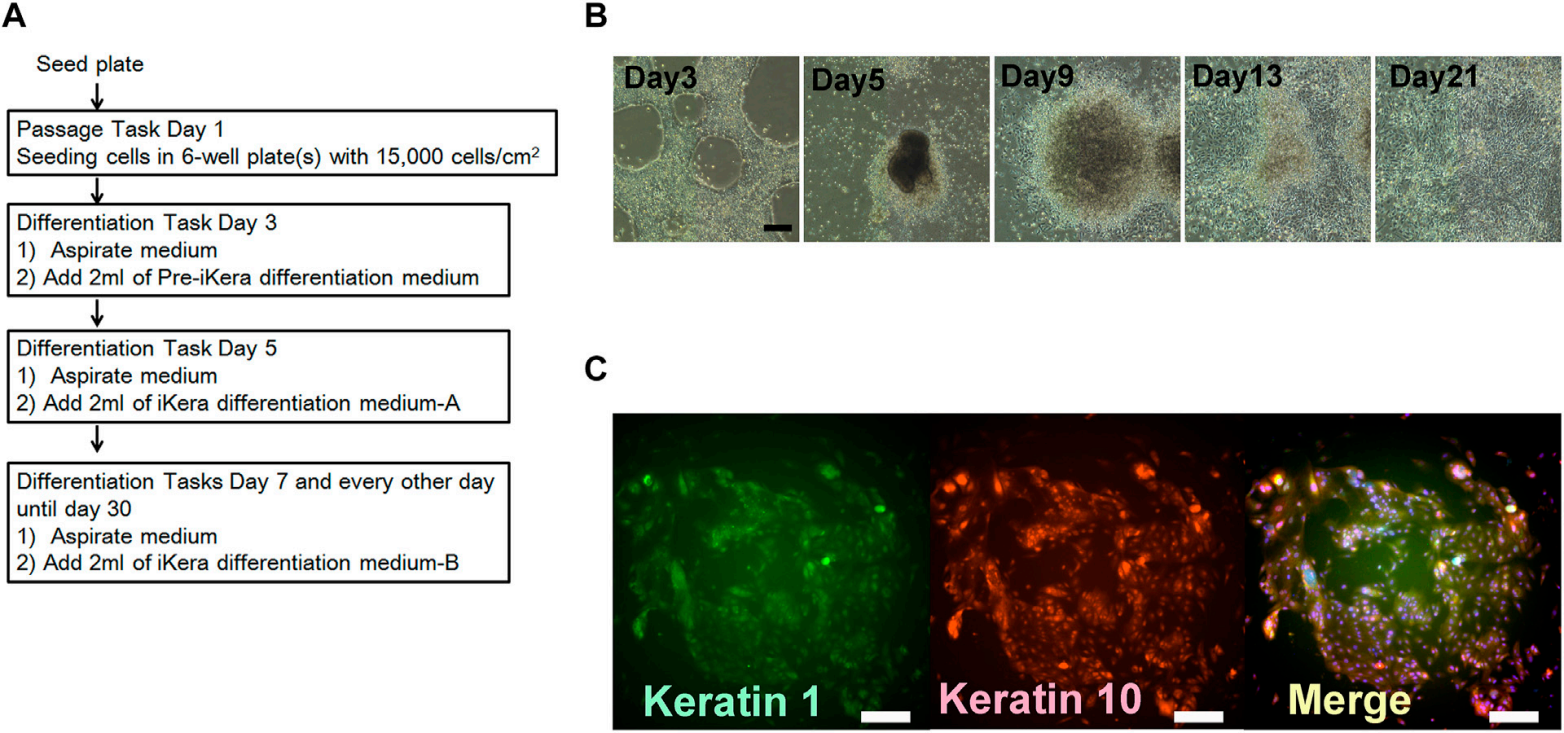
Keratinocyte differentiation using the human iPSC (253G1). **(A)** Schedule of the combination of processes including passage and differentiation. **(B)** Step by step changes in the cell figures based on phase contrast micrography. Scale bar = 200 μm. **(C)** Immunohistochemical staining for Keratin-1: green, and Keratin-10: red. The nucleus was stained with DAPI. Scale bar = 200 μm.

By switching the order from the maintenance task to each of the cardiomyogenic, hepatogenic, neurogenic, and keratinogenic differentiation task, the machine can seed cells into 6-well plates at the provided cell densities. Herein, the cells were sequentially treated with the pre-mixed differentiation media as shown in Figures 3A, 4A, 5A, 6A, 7A, respectively. The tasks performed for twelve to 14 days resulted in the appropriate differentiation of cells. During the processes, the cell features were markedly altered (Figures 3B, 4B, 5B, 6B, 7B, respectively). The differentiated cell clusters were fixed and employed for immunohistochemical analyses (Figures 4C, 5C, 6C-D, 7C, respectively), and confirmed the each aimed cell-type specific differentiations.

## 4 Discussion

We terminated the use of humanized multi-axes articulated arm and adopted the x-y-z-axes-rail-system, which enabled quicker cell-/medium-/plate-handling. The x-y-z-axes-rail-system can shorten the run-time from task to task, thereby preventing cells from dying *via* drying during the process. Moreover, the x-y-z-axes-rail-system can markedly decrease the efforts of engineers in modifying programs for machine movements, ultimately enabling quicker optimization to meet new experimental needs. Adoption of the new x-y-z-axes-rail system cut initial cost by approximately 10 thousand US dollars. Anticipated labor costs for program modifications are expected to be reduced by about 13%. Our automated culture machine is made with a combination of unit parts and driver programs available from the general markets, enabling quicker upgrades of the instruments with lower efforts and costs. The ability to perform quick upgrades of whole machines is a strong advantage and enables users to stay up-to-date on the latest state of the art iPSC technologies.

We demonstrated five continuous passages which can stably expand iPSC 625 ± 93 fold in 16 days. Although our proof of endurance for cell maintainance time was shorter than our previous demonstration, we found that cell-expansion efficiency was superior to that observed in the previous demonstration using feeder cells (Konagaya et al., 2015). We succeeded at a seamless transition from maintenance to differentiation tasks without the direct assistance of a human operator for the whole process (i.e., from expansion of the iPSC to differentiation into various types of somatic cells). Accordingly, researchers that use this machine can utilize iPSC-derived somatic cells without any experience or knowledge for the whole process of stem cell maintenance and differentiation. Therefore, it is very important for us to share not only the machine but also the task programs to ensure inter-institutional reproducibility and documentable information in articles.

Theoretically, human iPSC-derived differentiated somatic cells can replace the use of most animals and cell lines. However, such implementation is quite rare in research. In fact, only few pharmaceutical, food, and cosmetic discoveries have been achieved using iPSC-derived somatic cells as tools; this is because it is impossible to standardize the differentiation protocol to obtain the same cells from human iPSCs *via* manual handling and achieve inter-person reproducibility. Even among highly skilled scientists, experimental replication is hard to achieve. Moreover, it would be harder to guarantee the equality of iPSC-derived cells from different batches without any reliable process control. In this investigation, we obtained various types of somatic cells from human iPSC, independent of machine operators. Once human skills can be converted into machine process programs, stochastic changes between repetitions can be theoretically minimized. Accordingly, sharing this machine and the process programs can guarantee reproducibility and open a new era in various research fields.

Robots that aim to perfectly mimic human beings, such as LabDroid Maholo (Sasamata et al., 2021), will replace human laboratory bench work in the future. However, a long time is still required to achieve more effectiveness, besides the associated costs (McClymont and Freemont, 2017). Cost effectiveness is important for increasing the number of users of machines. We hope to not only be a presenter but also a hub for user networks to share accumulated knowledge. We believe that “sharing” will facilitate further social implementation of human iPSC. Our automated culture machine is not a humanized robot but could serve as a realistic choice for effective collaboration of machines and humans in various laboratories.

## Data Availability

The original contributions presented in the study are included in the article/Supplementary Material, further inquiries can be directed to the corresponding author.

## Supplementary material

SUPPLEMENTARY FIGURE S1 Structural differences between previous and current version culture machines. **(A)** Current version. **(B)** Previous version.

SUPPLEMENTARY FIGURE S2 Equivalency of automatically and manually processed cell number counting. Automatically and manually counted cell numbers of the same cell-suspended solutions (*n*=3). The linear approximation and correlation (R^2^) were calculated using Microsoft Excel.

SUPPLEMENTARY FIGURE S3 Internal alkaline phosphatase activity in machine-passaged cells. Cells were fixed with 1% para-formaldehyde at 25°C for 20 min. After several washes with pure water, cells were treated with ready-to-use BCIP-NBT reaction mixture (Nacalai Tesque Inc., Kyoto, Japan) at 25°C for 60 min. Cells containing activity were stained blue. Scale bar = 20 μm.

SUPPLEMENTARY MOVIE S1 Full (No-cut) movement of the machine during the “passage task.” The movement was shown in the actual speed.

SUPPLEMENTARY TABLE S1 The all primary and secondary antibodies used in this study are listed.

SUPPLEMENTARY TABLE S2 Comparisons of processing speed between human-operators, and previous and new versions of the automatic culture machine. The yellow frame indicates that the new version can finish a given task faster than the previous version. The orange frame indicates that the automated machine can finish faster than a human operator.
