# Supplementary material for: Compact automated culture machine for human induced pluripotent stem cell maintenance and differentiation
Source: Front Bioeng Biotechnol. 2022 Nov 29;10:1074990. doi: 10.3389/fbioe.2022.1074990 (PMC9744792; doi:10.3389/fbioe.2022.1074990)
Supplement: Supplementary file 1 [file Table1.pdf]

Supplementary Table S1

Target cell types	Antigen Name	Host	Clonality	Manufacturer	I.D.	Dilution
iPSC	Oct3/4	Mouse	monoclonal	BD	611202	1:100
	SSEA-4	Mouse	monoclonal	Millipore	MAB4304	1:100
	TRA 1-81	Mouse	monoclonal	Millipore	MAB4381	1:100
Cardiomyocytes	TnT	Rabbit	monoclonal	Abcam	ab92546	1:100
	Actinin	Mouse	monoclonal	Abcam	ab9465	1:100
Hepatocytes	Albumin	Rabbit	polyclonal	Dako	A0001	1:100
	HNF-4A	Goat	polyclonal	Santacruz	6556	1:100
Neuronal cells	Nestin	Mouse	monoclonal	Santacruz	23927	1:100
	βIII Tubulin	Mouse	monoclonal	Promega	G712A	1:100
	Neurofilament	Rabbit	polyclonal	Chemicon	AB1987	1:100
Keratinocytes	Keratin 1	Mouse	monoclonal	Santacruz	376224	1:100
	Keratin 10	Rabbit	polyclonal	BioLegend	19054	1:100

	Product name	host	clonality	Manufacturer	I.D.	Dilution
2nd Antibodies	mouse IgG(H+L) AlexaFluo488	Donkey	polyclonal	Thermo Scientific	A21202	1:300
	rabbit IgG(H+L) AlexaFluo488	Donkey	polyclonal	Thermo Scientific	A21206	1:300
	rabbit IgG(H+L) AlexaFluo546	Donkey	polyclonal	Thermo Scientific	A10040	1:300
	goat IgG(H+L) AlexaFluo546	Donkey	polyclonal	Thermo Scientific	A11056	1:300
