# Supplementary material for: Compact automated culture machine for human induced pluripotent stem cell maintenance and differentiation
Source: Front Bioeng Biotechnol. 2022 Nov 29;10:1074990. doi: 10.3389/fbioe.2022.1074990 (PMC9744792; doi:10.3389/fbioe.2022.1074990)
Supplement: Supplementary file 2 [file Table2.pdf]

Supplementary Table S2

	Human operator			Automated machine					
Task	Beginner	Trained	Expert	Process	Old version		New version (this study)		Time saving
**Preparation**	5.5	2.1	1	dispense PBS in to three dishes	12.56	54.12	11.59	44	10.12
				preparation of culture medium for three dishes	9.22		8.16		
				taking out dish from incubator	4.19		3.36		
				wash and exchange medium	22.1		15.52		
				transfer the dish to incubator	3.05		2.43		
				discard the used tubes	2.21		1.34		
**Passage**	50.5	34.5	27.2	dispense PBS	4.25	59.58	3.41	50.68	8.9
				preparation of mediums for three dishes	15.38		13.46		
				taking out dish from incubator	1.08		1.1		
				transfer the dish to incubator	3.07		2.4		
				taking out dish from incubator	1.08		1.1		
				detachment of the cells, and preparation of dispersed cell solution	19.02		16.52		
				seeding	10.3		8.26		
				transfer the dish to incubator	3.07		2.4		
				discard dishes and tubes	2.33		2.03		
**Cell count**	9.2	7	4.1		No function		3.8	3.8	–

(minutes)
