# Supplementary figures and images for: Compact automated culture machine for human induced pluripotent stem cell maintenance and differentiation
Source: Front Bioeng Biotechnol. 2022 Nov 29;10:1074990. doi: 10.3389/fbioe.2022.1074990 (PMC9744792; doi:10.3389/fbioe.2022.1074990)

**Supplementary Figure 2**

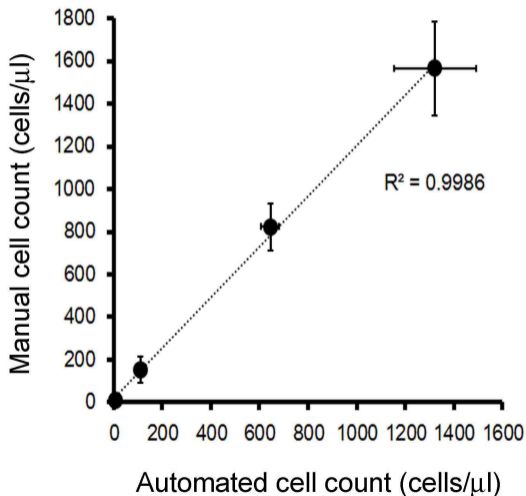

Supplement: Supplementary file 3 [file Image2.pdf]

## Supplementary Figure 3

(A)

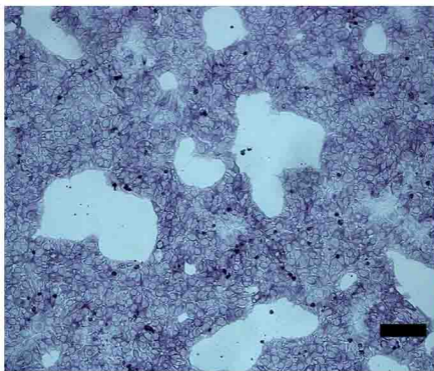

(B)

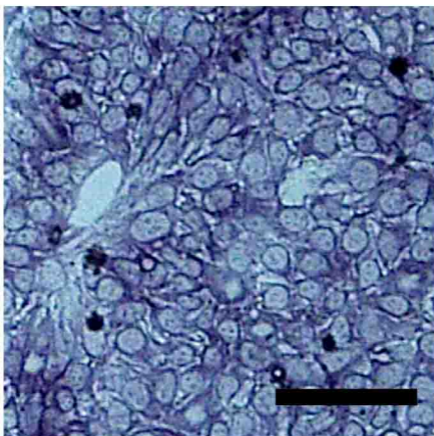

Supplement: Supplementary file 4 [file Image3.pdf]
