# Supplementary material for: Compact automated culture machine for human induced pluripotent stem cell maintenance and differentiation
Source: Front Bioeng Biotechnol. 2022 Nov 29;10:1074990. doi: 10.3389/fbioe.2022.1074990 (PMC9744792; doi:10.3389/fbioe.2022.1074990)

# Supplementary Figure 1

## (A)

### New version: x-y-z-axes-rail-system

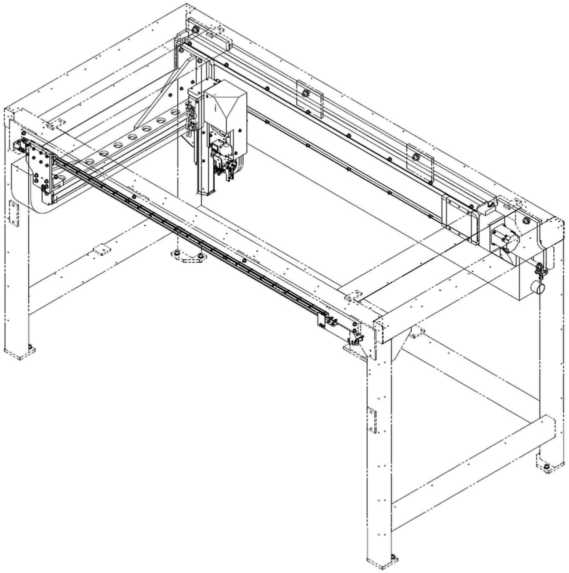

## (B)

### Old version: 6-axes articulated-robotic arm

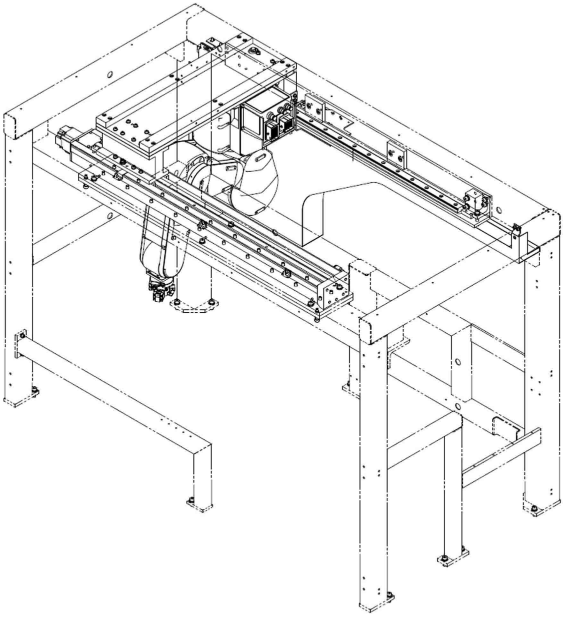

Supplement: Supplementary file 7 [file Image1.pdf]
